# Usefulness of Combining Galectin-3 and BIVA Assessments in Predicting Short- and Long-Term Events in Patients Admitted for Acute Heart Failure 

**DOI:** 10.1155/2014/983098

**Published:** 2014-06-30

**Authors:** Benedetta De Berardinis, Laura Magrini, Giorgio Zampini, Benedetta Zancla, Gerardo Salerno, Patrizia Cardelli, Enrico Di Stasio, Hanna K. Gaggin, Arianna Belcher, Blair A. Parry, John T. Nagurney, James L. Januzzi, Salvatore Di Somma

**Affiliations:** ^1^Emergency Medicine, Department of Medical-Surgery Sciences and Translational Medicine, Sapienza University of Rome, Sant'Andrea Hospital, Via di Grottarossa 1035/1039, 00189 Rome, Italy; ^2^Clinical and Molecular Medicine Department, Sapienza University of Rome, Sant'Andrea Hospital, 00189 Rome, Italy; ^3^Institute of Biochemistry and Clinical Biochemistry, Catholic University of the Sacred Heart, 00168 Rome, Italy; ^4^Division of Cardiology, Massachusetts General Hospital, Harvard Medical School, Boston, MA 02114, USA; ^5^Department of Emergency Medicine, Massachusetts General Hospital, Harvard Medical School, Boston, MA 02114, USA

## Abstract

*Introduction*. Acute heart failure (AHF) is associated with a higher risk for the occurrence of rehospitalization and death. Galectin-3 (GAL3) is elevated in AHF patients and is an indicator in predicting short-term mortality. The total body water using bioimpedance vector analysis (BIVA) is able to identify mortality within AHF patients. The aim of this study was to evaluate the short- and long-term predictive value of GAL3, BIVA, and the combination of both in AHF patients in Emergency Department (ED). *Methods*. 205 ED patients with AHF were evaluated by testing for B type natriuretic peptide (BNP) and GAL3. The primary endpoint was death and rehospitalization at 30, 60, 90, and 180 days and 12 and 18 months. AHF patients were evaluated at the moment of ED arrival with clinical judgment and GAL3 and BIVA measurement. *Results*. GAL3 level was significantly higher in patients >71 years old, and with eGFR < 30 cc/min. The area under the curve (AUC) of GAL3 + BIVA, GAL3 and BIVA for death and rehospitalization both when considered in total and when considered serially for the follow-up period showed that the combination has a better prognostic value. Kaplan-Meier survival curve for GAL3 values >17.8 ng/mL shows significant survival difference. At multivariate Cox regression analysis GAL3 is an independent variable to predict death + rehospitalization with a value of 32.24 ng/mL at 30 days (*P* < 0.005). *Conclusion*. In patients admitted for AHF an early assessment of GAL3 and BIVA seems to be useful in identifying patients at high risk for death and rehospitalization at short and long term. Combining the biomarker and the device could be of great utility since they monitor the severity of two pathophysiological different mechanisms: heart fibrosis and fluid overload.

## 1. Introduction

In patients with acute heart failure (AHF), the occurrence of rehospitalization and death is very common [1, 2]. There is need for tools to immediately identify patients with AHF at high risk for short- and long-term mortality and readmission. Galectin-3 (GAL3) is a *β*-galactoside-binding lectin overexpressed by macrophages during phagocytosis that has been shown to be elevated in patients with AHF representing a prognostic biomarker for future adverse events such as death and rehospitalization [3, 4]. The adverse outcome in patients with elevated circulating level of GAL3 has been linked with the presence of enhanced amount of the fibrosis of the heart [5]. Between patients that need to be hospitalized for AHF the occurrence of body congestion is a common finding [6].

Recently, the assessment of total body water using bioimpedance vector analysis (BIVA) has been suggested to be useful for the differential diagnosis of dyspnea, identifying patients with fluid overload [7]. Moreover, within subjects referring to the Emergency Department (ED) for AHF, BIVA has been demonstrated to be able to identify people at high risk for short-term mortality [8, 9]. In consideration of the two different aspects of GAL3 and of BIVA (the first is a fibrosis marker, and the second is a dynamic marker of congestion), we decided to evaluate in this study the degree of congestion correlated to that of fibrosis in AHF patients alone or together for the prediction of events. So far no data are available on potential usefulness of combining GAL3, as a biomarker of heart fibrosis, and BIVA, as a device for detecting fluid overload in patients with AHF in order to identify subject at high risk for future adverse outcome. The aim of this study was to evaluate the short- and long-term predictive value of GAL3, BIVA, and the combination of both in patients with AHF at the moment of their hospital admission.

## 2. Materials and Methods

### 2.1. Study Design

In a prospective, blinded international study, patients presenting to ED with AHF were evaluated by testing for B type natriuretic peptide (BNP) and GAL3. The primary endpoint was death and rehospitalization at 30, 60, 90, and 180 days and 12 and 18 months.

We enrolled 205 subjects from March 2012 to September 2013 at two tertiary care academic medical centers members of the Global Research on Acute Conditions Team (GREAT): Sant'Andrea Hospital (Rome, Italy) and the Massachusetts General Hospital (Boston, MA). All study procedures were approved by local institutional review boards. AHF patients were evaluated at the moment of ED arrival with clinical judgement and blood routine laboratory tests plus GAL3 and BIVA. Inclusion criteria included moderate or severely symptomatic AHF (classified on the basis of current guidelines [10]) requiring intensification of diuretic therapy [10]. Exclusion criteria included renal failure requiring current renal replacement therapy, ≥8 hours from the first dose of intravenous diuretic, and unwillingness or inability to participate in study procedures. At the moment of ED arrival, baseline demographics, vital signs, and results of physical examination were evaluated and recorded after informed consent was signed. The protocol was designed following the criteria of the Declaration of Helsinki and was approved by the ethical committee of each participating hospital. Peripheral venous blood was withdrawn and processed as noted below.

### 2.2. Blood Analysis

Peripheral venous blood was withdrawn by each patient and put into tubes containing ethylenediaminetetraacetic acid or no anticoagulant and spun for 15 minutes; samples were immediately aliquoted to freezer tubes and frozen at −80° for biomarkers measurement following the completion of the trial. Samples were thawed for the first time for measurement of biomarkers. Biomarkers of myocardial stretch BNP (Alere Triage BNP, San Diego, CA), biomarkers of risk stratification of patients GAL3 (VIDAS, Biomerieux, Marcy l'Étoile, France), and biomarkers of renal function included blood urea nitrogen (BUN), serum creatinine, and estimated glomerular filtration rate (eGFR, estimated using the simplified modification of diet in renal disease equation [11]). Clinicians were blinded to GAL3 and BIVA results.

### 2.3. Galectin-3

GAL3 (VIDAS, Biomerieux, Marcy l'Étoile, France) is a quantitative, one-step sandwich assay with fluorescence detection, designed for use with the VIDAS automated immunoassay system. Briefly the system measures GAL3 in human serum or plasma (200 *μ*L) using the ELFA (enzyme-linked fluorescent assay) technique in 20 minutes. All stages of the assay are performed automatically by the instrument, calculating the concentration of GAL3 relative to a stored calibration curve and enabling patients to be assigned as low, intermediate, or high risk of GAL3 mediated HF. Correct assay performance and validation of results are ensured by analysis of the control sample included in the kit. Decisional cut-offs are represented by ≤17.8 ng/mL “low risk,” 17.8–25.9 ng/mL “intermediate risk,” and ≥25.9 ng/mL “high risk” [12]; the three risk categories have been previously defined for the BGM Galectin-3 microplate assay [13].

### 2.4. BIVA Assessment

We used standard tetrapolar bioelectrical impedance electrodes at a frequency of 50 kHz (Akern Srl, Pontassieve, Florence, Italy). The BIVA measurement assessed at patients' ED arrival was performed at bedside, with the patient supine, without metal contacts, and with inferior limbs at 45° and superior limbs abducted at 30° to avoid skin contacts. Four skin electrodes were applied (two on the wrist and two on the ipsilateral ankle) maintaining a minimal interelectrode distance of 5 cm. The machine used an alternating current flux of 300 *μ*A and an operating frequency of 50 kHz. The results were visualized in two ways: as a vector or as a BIVA-derived hydration percentage. The first method includes a direct impedance plot which measures resistance (Rz) and reactance (Xc) as a bivariate vector in a nomogram. Reference values adjusted for age, BMI, and gender are plotted as tolerance ellipses in the same coordinate system. Three tolerance ellipses are distinguished, corresponding to the 50th, 75th, and 95th vector percentiles of the healthy reference population. The major axis of this ellipse indexes hydration status and the minor axis reflects tissue mass. The second method involves a scale called a hydrograph (or hydrogram), which expresses the state of hydration as a percentage (HI). This value is calculated by an independently determined equation that uses the two components of BIVA, Rz and Xc. The normal value is 73.3% with tolerance between 72.7% and 74.3%, corresponding to the 50th percentile. On arrival at the ED, Rz and Xc were recorded, normalized by the subject's height, and graphically expressed on the Rz-Xc plane; furthermore, HI was also assessed [8, 14–17]. Clinicians were blinded to the results of BIVA.

### 2.5. Follow-Up

In-hospital, 30-, 60-, 90-, and 180-day and 12- and 18-month follow-up events (deaths or rehospitalization) were recorded.

### 2.6. Statistical Analysis

Continuous variables were summarized as mean ± standard deviation (SD) if normally distributed and as median and interquartile range [IQR] if not normally distributed. Discrete variables are shown as percentage. Baseline variables of study participants for events (death + rehospitalization) and death were compared using Student's *t*-test for continuous variables or *X*_2_ test for discrete variables if data were normally distributed; the Mann-Whitney *U* test was used for continuous variables and Fisher's exact test for discrete variables in the states of nonnormality. To determine the prognostic value of GAL3 and phase angle, the receiver operating characteristic (ROC) tests compared the results of GAL3 and phase angle and combination of both for predicting rehospitalization or death, expressed as area under the curve (AUC); the *P* value was obtained. Univariable comparisons between baseline characteristics were used to identify candidate variables for entry to a multivariable logistic regression model in order to select the variables most predictive of patients' outcomes; only those with a *P* value <0.05 were retained for multivariable modeling. Net reclassification index (NRI) analysis was used to improve the accuracy of the risk-prediction model for in-hospital mortality. All statistical analyses were performed using Medcalc version 12.1.4 (Medcalc Software, Mariakerke, Belgium) software. All *P* values are two-sided with a value of <0.05 considered significant.

## 3. Results

**Figure 1:**
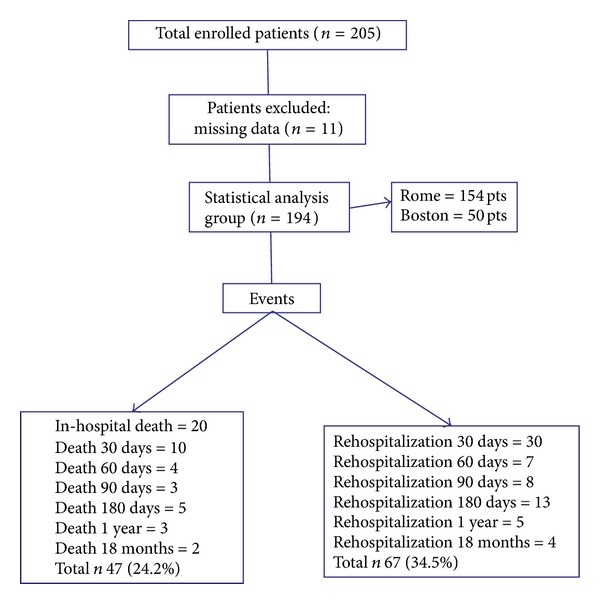
Flowchart of the study.

**Figure 2:**
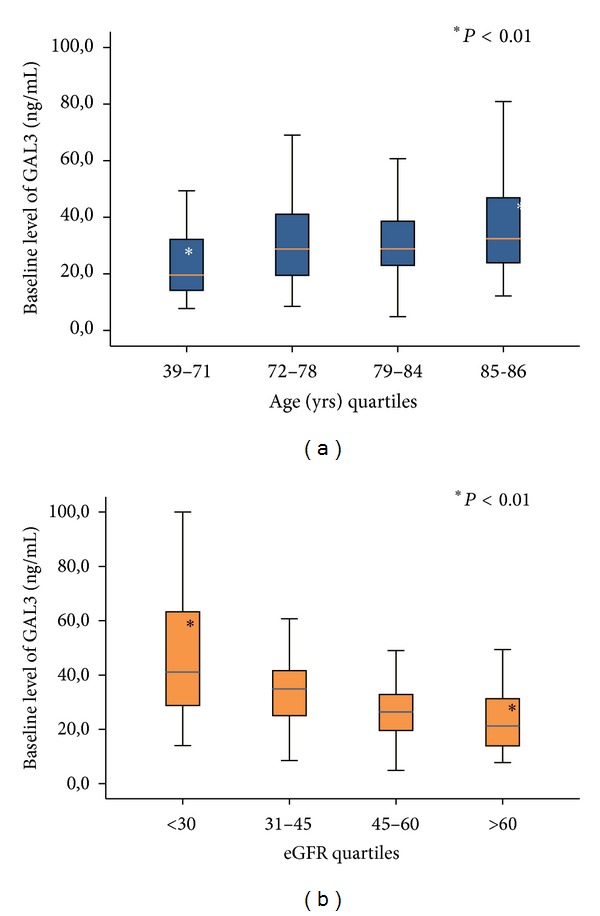
GAL3 levels increase proportionally with age quartiles (a) and decrease with eGFR quartiles (b).

**Figure 3:**
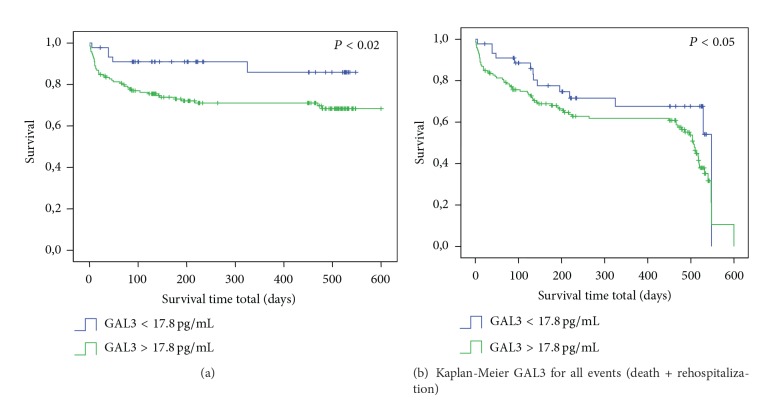
Kaplan-Meier for death (a) and events (b) on the basis of GAL3 values greater than 17.8 ng/mL.

Figure 1 showed the flowchart of the study, 11 patients were excluded for missing data, total deaths in hospital and during 18-month follow-up were 47 (24.2%), and total rehospitalization during all follow-up period was 67 (34.5%). Patients' characteristics are showed in Table 1. At the moment of ED arrival compared to survivors there were no statistically significant differences for demographic data in patients with death or total adverse events (rehospitalization and death) observed in all periods of the study. Beta blockers use was higher in patients who survived. On the contrary, in patients who died, there was a significant increase of serum creatinine (sCr), blood urea nitrogen (BUN), and white blood cells (WBC). When considering the combination of death and rehospitalization during follow-up period, there was significant increase of age, hypertension, diabetes mellitus, use of ACE inhibitors (ACEi), sCr, and BUN values in patients who develop events.  Table 2 shows that, compared to survivors, GAL3 level at admission was significantly higher in both groups that died or that developed death + rehospitalization during follow-up. On the contrary, BNP value was not different within groups of patients who survived, died, or were rehospitalized. As for BIVA data, there was a significant increase of Xc in patients who died during follow-up compared to survivors (Table 2).  Figure 2 shows the value of GAL3 subdivided in quartiles on the basis of age (a) and of eGFR (b).  Figure 2(a) demonstrates that GAL3 level was significantly higher in patients within quartiles of age >71 years and in patients within quartiles of eGFR<30 mL/min/1.73 m^2^ (Figure 2(b)).  Table 3 shows the ROC curve analysis of GAL3, BIVA (phase angle), and GAL3 + phase angle for mortality and rehospitalization for 30, 60, 90, and 180 days and 12 and 18 months.  Figure 3 shows the Kaplan-Meier survival curve for death (a) and for total events (death and rehospitalization) (b) on the basis of GAL3 values greater than 17.8 ng/mL, international cut-off [12]. It is evident that in patients with GAL3 >17.8 ng/mL there was a higher incidence of death or of all events (death and rehospitalization) during 18-month follow-up. Cox regression analysis demonstrated that GAL3 is an indipendent variable to predict death to predict death and rehospitalization with a value of 32.24 ng/mL at 30 days (*P* < 0.005). We constructed a clinical model based on variables suggested by the referee (age, sex, BNP, LVEF, and creatinine), using as decisional cut-off of the median values of our entire population. Each patient was classified at low or high risk for development of events on the basis of the positivity at the different predictive variables. The best predictive model in terms of sensitivity and specificity was obtained with positivity at 3 out of 5 variables (sensitivity 61.4%, specificity 61.8%, and accuracy 62%). The use of galectin-3 at a threshold of 17.8 combined to previously described clinical model was able to improve the global risk classification (NRI) of 18% (NRI for events +27%, NRI for no events −9%, *P* = 0.021). The use of galectin-3 and BIVA was able to get a NRI of 20% especially improving the “no events” correct reclassification (NRI for events +16%, NRI for no events +4%, *P* = 0.012).

## 4. Discussion

Cardiac remodeling and congestion are crucial determinants of the clinical outcome of heart failure (HF) and are linked to disease progression and poor prognosis [18]. Recently it has been demonstrated that slowing or reversing the progression of remodeling could become a therapeutic goal of HF patients' management. Circulating plasma concentrations of BNP is currently the most commonly used biomarker in AHF and its level is generally increased in proportion to the severity of the myocardial stretch or overload [19]. However, the applicability of BNP is limited, since its levels substantially vary over the day, and is not related to the underlying cardiac disease process. Prognostic value of GAL3 when upregulated in hypertrophied hearts has been confirmed in a number of studies [2, 3, 20–25]. Data from our study strongly support the original research papers that showed how GAL3 could play an important role in the underlying structural heart disease processes. Elevated value of GAL3 is associated with heart failure progression due to increase of heart fibrosis leading to poor outcome in AHF patients. From our results GAL3 value in patients who develop higher incidence of death and rehospitalization was independent risk factor with a cut-off of 32.24 ng/mL greater than the cut-off of 17.8 ng/mL that is currently accepted, Table 4 [12, 20–26]. Moreover GAL3 was statistically higher in patients who died (*P* < 0.001) and were rehospitalized compared to survivors (*P* < 0.001). Our results are also in agreement with van Kimmenade et al. who demonstrated that, elevated GAL3 levels in patients presenting to ED for AHF, GAL3 was the best independent predictor for 60-day mortality [4]. Furthermore our results are in the same directions with the ones from Shah et al. who demonstrated that in patients with AHF presenting to ED a value of GAL3 levels above the median value had higher incidence of mortality [3]. Our study is probably the first in which a long-term follow-up for mortality and rehospitalization has been studied in AHF patients in consideration of GAL3 levels measured in ED; furthermore, our follow-up study was serially conducted (30-, 60-, 90-, and 180-day and 12- and 18-month follow-up events (deaths or rehospitalization)). In literature there are very few data on short- and long-term follow-up but all these papers are not serially [3, 4, 27]. Recently published data from three large research trials from HF patients. They demonstrated that elevation of GAL3 levels was significantly predictive of rehospitalization of 30 days from discharge. Moreover those patients of elevation of GAL3 at the time of hospitalization were readmitted within 30 days at three times of rate of patients without GAL3 elevation and the increased risk of hospitalization conferred by GAL3 elevation persisted at 60, 90, and 120 days in the study [28].

As described in literature GAL3 was able to identify those AHF patients at risk for short-term death or for the combination of death and readmission [4]; in our study GAL3 measured at the moment of ED arrivals predicts adverse events better than BNP. So we can consider that on the basis of this data BNP has not a real prognostic value at admission; our group already demonstrated this finding in the ITALIAN RED study [29]. However, we must underline the fact that BNP is internationally considered as the gold standard biomarker in any risk score of AHF patients.

We also analyzed the levels of GAL3 based on eGFR quartiles and we demonstrated that GAL3 levels were significantly higher in patients with reduced eGFR (<30 mL/min/1.73 m^2^). O'Seaghdha et al. already demonstrated that high levels of GAL3 were associated with a rapid decline of eGFR and with a higher risk of incidence of chronic kidney disease (CKD) [30]. Findings from our study are also supported by a study from Tang et al. who recently demonstrated that high GAL3 levels are associated with a poor renal function [24]. As a consequence, the inverse relationship between GAL3 and renal function, which we observed in our AHF patients, leads to the suggestion that increased plasma GAL3 in AHF might be linked to renal dysfunction, and the ability of GAL3 to predict outcomes in HF might reflect the consequences of renal impairment [3, 24, 25]. Moreover, GAL3 levels were significantly higher in patients with age >71 years, too. This suggests that in evaluating the value of GAL3 the physician should take into account the age of patients as confounding factor. This association of elevated GAL3 level and age should not be surprising; it is well known that heart fibrosis is a structural process related to aging. Other authors already found this relationship with GAL3 and age in the same cohort of patients [24]. We also found that BIVA had significant prognostic value for death for each considered follow-up time. This is not surprising and confirms previous study of our group, we also demonstrated additive value for AHF risk stratification of BIVA + BNP [8, 9]. The most important of our opinion results of our study is that the combination of BIVA + GAL3 increases the prognostic value for death and also for rehospitalization. It seems that the prognostic value of the combining of BIVA and GAL3 is of great significance since they mirror two different physiopathological aspects of HF. BIVA (phase angle) represents the degree of body fluid congestion, typical of AHF patients especially in the end stage of the disease; on the other hand, GAL3 value represents the level of fibrosis and the heart remodeling [31]. The combination of BIVA (phase angle) and GAL3 seems to have the better AUC compared to BIVA alone or GAL3 alone in predicting patients' adverse outcome. Nevertheless in our study multivariate Cox regression analysis showed that GAL3 is an independent variable more than BIVA variables to predict death and rehospitalization of value 32.24 ng/mL only at 30 days. These results strengthen the hypothesis that GAL3 could be considered a better predictive marker in comparison to BIVA and BNP when measured at admission in ED. More studies on larger population group are needed to confirm this hypothesis. When the survival Kaplan-Meier curve was made for GAL3, we found that, considering the cut-off of 17.8 ng/mL, those patients above this cut-off had a significant higher incidence of death and rehospitalization. This confirms the importance of cut-off of GAL3 of 17.8 ng/mL representing an additive value to help physicians especially emergency physicians to individuate those patients of adverse outcomes.

## 5. Conclusion

In patients admitted for AHF an early assessment of GAL3 and BIVA seems to be useful in identifying patients at high risk for death and rehospitalization at short and long term. Combining the biomarker and the device could be of great utility since they are monitoring the severity of two pathophysiological different mechanisms underlining the severity of disease: heart fibrosis and fluid overload. GAL3 per se has the strongest independent predicting value for death and rehospitalization at 30 days with a cut-off of 32.24 ng/mL. The use of combining GAL3 and BIVA assessment, at the moment of AHF patients' presentation, could support decision making and different therapeutic approach.

## Figures and Tables

**Table 1:** Baseline characteristics of study subjects as a function of the subsequent development of events (in-hospital death and rehospitalization and death at 18-month follow-up) and total death.

	Total	Events		*P*	Death		*P* value
	(*N* = 194)	Yes (*N* = 88)	No (*N* = 106)		Yes (*N* = 47)	No (*N* = 147)	
	*n* (%)	*n* (%)	*n* (%)		*n* (%)	*n* (%)	
Demographics							
Age (years) (mean, SD)	76.23 ± 10.84	78.23 ± 10.58	74.51 ± 10.92	0.01	80.09 ± 10.54	74.97 ± 10.74	0.08
Male	107 (56.1%)	48	59	0.37	24 (12.6%)	83 (43.6%)	0.41
Medical history							
Prior heart failure	104 (54.73%)	47	57	0.42	24 (12.63%)	80 (42.10%)	0.56
CKD	58 (30.52%)	28	30	0.42	16 (8.42%)	42 (22.10%)	0.54
Hypertension	149 (78.42%)	64	85	0.05	32 (16.84%)	117 (61.5%)	0.47
Myocardial infarction	63 (33.15%)	29	34	0.54	15 (7.89%)	48 (25.26%)	0.83
CAD	67 (35.26%)	28	39	0.22	11 (5.78%)	56 (29.47%)	0.50
COPD	52 (27.36%)	31	33	0.39	17 (8.94%)	47 (24.73%)	0.67
Atrial fibrillation	79 (41.57%)	37	42	0.51	19 (10.0%)	60 (31.57%)	0.85
Diabetes mellitus	86 (45.26%)	47	39	0.02	22 (11.5%)	64 (33.6%)	0.80
Prior medication							
ACE-i	54 (28.42%)	31	23	0.03	15 (7.89%)	39 (20.52%)	0.57
ARBs	26 (13.68%)	8	18	0.07	5 (2.63%)	21 (11.05%)	0.47
BB	115 (60.52%)	45	70	0.01	15 (7.89%)	100 (52.63%)	<0.001
Loop diuretic	124 (65.26%)	57	67	0.52	30 (15.78%)	94 (49.47%)	0.72
Nitrates	60 (31.57%)	25	25	0.29	13 (6.84%)	37 (19.47%)	0.84
Digoxin	20 (10.52%)	9	11	0.56	7 (3.68%)	13 (6.84%)	0.27
ASA	96 (50.52%)	43	53	0.45	18 (9.47%)	78 (41.05%)	0.43
NSAID	19 (10.0%)	11	8	0.19	2 (1.05%)	17 (8.94%)	0.12
Spironolactone	24 (12.63%)	13	20	0.27	7 (3.68%)	26 (13.68%)	0.58
Jugular venous distention	68 (35.78%)	31	37	0.52	17 (8.94%)	51 (26.84%)	0.87
Lower extremity edema	106 (55.78%)	46	60	0.22	21 (11.05%)	85 (44.73%)	0.77
Cough	39 (20.52%)	17	22	0.42	7 (3.68%)	32 (16.84%)	0.27
Orthopnea	136 (71.57%)	67	69	0.12	34 (17.89%)	102 (53.68%)	0.89
Paroxysmal nocturnal dyspnea	88 (46.31%)	46	42	0.08	26 (13.68%)	62 (32.63%)	0.15
Chest pain	40 (21.05%)	16	24	0.23	7 (3.68%)	33 (17.36%)	0.23
Hepatojugular reflux	41 (21.57%)	25	16	0.02	7 (14.21%)	14 (7.36%)	0.11
Murmur	84 (44.21%)	39	47	0.46	22 (11.57%)	64 (33.68%)	0.80
Rales	149 (78.42%)	72	77	0.19	42 (22.10)	107 (56.31%)	0.36
Wheezing	145 (76.31%)	21	24	0.54	9 (4.73%)	36 (18.94%)	0.39
Edema	115 (60.52%)	58	57	0.16	28 (14.73%)	87 (45.78%)	0.87
Interstitial edema	120 (63.15%)	59	61	0.18	34 (17.89%)	86 (45.26%)	0.13
Pleural effusions	99 (52.10%)	46	53	0.54	28 (14.73%)	71 (37.36%)	0.23
Heart rate	88.14 ± 24.70	88.32 ± 24.64	87.28 ± 25.06	0.77	93.66 ± 25.74	85.81 ± 24.26	0.59
SBP	146.94 ± 33.67	147.89 ± 34.53	147.46 ± 32.81	0.93	145.1 ± 33.0	148.5 ± 33.78	0.75
DBP	79.30 ± 17.64	79.26 ± 16.62	79.57 ± 18.56	0.90	78.55 ± 15.05	79.71 ± 18.45	0.31
sCr (mg/dL)	1.56 ± 1.09	1.73 ± 1.26	1.34 ± 0.81	0.01	1.99 ± 1.49	1.37 ± 0.8	0.001
BUN (mg/dL)	34.9 ± 21.5	39.21 ± 22.54	30.28 ± 18.52	0.003	44.0 ± 23.8	31.27 ± 18.09	0.005
eGFR	55.0 ± 28.5	50.99 ± 29.71	59.40 ± 25.68	0.03	45.48 ± 29.01	58.80 ± 26.76	0.48
Na (mE/L)	136.7 ± 64	135.97 ± 6.05	137.46 ± 4.93	0.62	134.26 ± 6.54	137.59 ± 4.88	0.16
Glucose	145 ± 70	151.94 ± 66.79	145.09 ± 74.60	0.51	159.6 ± 73.4	144.5 ± 70.0	0.55
WBC	9.8 ± 9.8	11.01 ± 11.77	9.27 ± 4.04	0.16	13.1 ± 15.6	9.05 ± 3.68	<0.001
HGB	11.9 ± 0	11.68 ± 2.02	12.15 ± 1.99	0.11	11.56 ± 2.09	12.05 ± 2.02	0.86
RDW	17.4 ± 0	16.79 ± 2.65	17.49 ± 16.13	0.68	17.23 ± 2.9	17.14 ± 13.65	0.61
BMI	28.95 ± 13.76	27.98 ± 8.32	29.28 ± 16.27	0.49	26.7 ± 4.02	29.3 ± 14.9	0.95

**Table 2:** Baseline biomarkers as a function of the primary endpoint of events (in-hospital death and rehospitalization and death at 18-month follow-up) and death.

Variable (mean ± SD)	Total (*N* = 194)	Events (death + rehospitalization)		*P*	Death		*P*
		Yes (*N* = 88)	No (*N* = 106)		Yes (*N* = 47)	No (*N* = 147)	
GAL3 (ng/mL)	32.19 ± 19.03	37.15 ± 21.8	26.12 ± 13.6	<0.001	40.58 ± 23.09	28.16 ± 15.96	<0.001
BNP (pg/mL)	872.9 ± 1024.4	969.5 ± 1205.5	774.5 ± 837.1	0.91	1030.0 ± 1059.9	821.25 ± 1014.6	0.27
Hydration (%)	79.44 ± 6.55	80.75 ± 6.55	78.75 ± 6.66	0.75	80.62 ± 7.6	79.05 ± 6.19	0.11
Xc/H	34.8 ± 16.4	27.0 ± 14.2	27.35 ± 11.63	0.93	28.25 ± 26.85	17.07 ± 11.28	0.05
Rz/H	364.25 ± 136.8	363.73 ± 140.02	341.07 ± 120.88	0.21	386.05 ± 340.54	155.30 ± 119.68	0.28
Phase angle	4.4 ± 1.7	4.3 ± 1.7	4.7 ± 1.5	0.17	4.24 ± 2.09	4.62 ± 1.48	0.79

**Table 3:** ROC curve analysis of GAL3, BIVA (phase angle), and GAL3 + phase angle for mortality and rehospitalization for 30, 60, and 90 days, 1 year, and 18 months.

	GAL3 + phase angle		GAL3		Phase angle	
	AUC	*P*	AUC	*P*	AUC	*P*
Rehospitalization						
30 days	0.526	ns	0.51	ns	0.52	ns
60 days	**0.625**	**0.003**	0.61	0.04	0.54	0.04
90 days	0.583	ns	0.57	ns	0.57	ns
180 days	**0.545**	**0.05**	0.54	ns	0.52	ns
12 months	0.52	ns	0.54	ns	0.53	ns
18 months	**0.620**	**0.04**	0.59	0.04	0.52	ns

Death						
30 days	**0.764**	**0.0001**	0.69	0.002	0.64	0.01
60 days	**0.754**	**0.0001**	0.68	0.0001	0.68	0.003
90 days	**0.667**	**0.005**	0.64	0.01	0.58	0.04
180 days	**0.841**	**0.0001**	0.67	0.006	0.79	0.0001
12 months	**0.833**	**0.0001**	0.72	0.002	0.79	0.0001
18 months	**0.863**	**0.0001**	0.73	0.0003	0.86	0.0001

**Table 4:** Multivariate Cox regression analysis GAL3 is an independent variable to predict death and rehospitalization with a value of 32.24 ng/mL at 30 days (*P* < 0.005).

Variables	Logistic regression			
	*B*	SE	Wald	*P*
GAL3	**0.671 **	**0.370 **	**3.290 **	**0.05 **
Phase angle	−1.462	0.773	3.574	<0.03
Hi	0.103	0.708	0.021	ns
